# Clinical study on the application of a 2-*μ*m continuous wave laser in transurethral vaporesection of the prostate

**DOI:** 10.3892/etm.2013.951

**Published:** 2013-02-05

**Authors:** YONG XU, DONGCHONG SUN, ZHITAO WEI, BAOFA HONG, YONG YANG

**Affiliations:** Department of Urology, PLA General Hospital, Beijing 100056, P.R. China

**Keywords:** 2-*μ*m continuous wave laser, benign prostatic hyperplasia, transurethral resection

## Abstract

The present study aimed to evaluate the method and clinical effects of transurethral dividing vaporesection of the prostate in the management of benign prostatic hyperplasia (BPH) using the RevoIix 70 W 2-*μ*m continuous wave (cw) laser. A total of 155 BPH patients were treated transurethrally under epidural or sacral anesthesia using the dividing vaporesection technique. Of these, 80 had a prostate volume of ≤80 ml and 75 had a prostate volume of >80 ml. Pre- and post-operative data were evaluated for prostate-specific antigens (PSAs), post-void residual volume (PVR), maximum urinary flow rate (Qmax), International Prostate Symptom Score (IPSS) and quality of life (QoL). Statistical analyses were performed using the SPSS 16.0 software. Treatment effectiveness evaluations were also conducted. In the ≤80 ml prostate volume group, the mean PSA level decreased from 3.8±0.9 to 2.6±1.3 ng/ml. The PVR, mean Qmax, IPSS and QoL score improved significantly (P<0.05) in each group but no statistically significant difference was identified between the two groups. With a shorter surgery duration, safe use and high cutting efficiency and rapid vaporization ability, the 2-*μ*m laser vaporesection technique shows superiority compared to standard techniques in the treatment of BPH.

## Introduction

Benign prostate hyperplasia (BPH) is one of the most common diseases affecting the health of aging males. The incidence rate of benign prostatic hyperplasia has been the highest in recent years and represents the aging trend (1). A study by Roehrborn *et al* recorded the histological prevalence of BPH based on a review of five studies relating age to histological findings in human male prostate glands (2). A palpable enlargement of the prostate has been identified in up to 20% of males in their 60s and in 43% of males in their 80s (3). Transurethral resection of the prostate (TURP) is presently the most common active treatment for BPH and has been established as the gold standard surgical procedure (4), however, the overall complication morbidity rate of TURP is comparatively high. To minimize the perioperative morbidity of TURP, various minimally invasive alternatives, including laser techniques, were introduced to clinical practice in an effort to reduce treatment-related complications (5). Compared with TURP, transurethral laser resection of the prostate (TULRP) has several advantages, including a lower incidence of complications, a minimal risk of bleeding or requirement for blood transfusion, a decreased risk of water intoxication and decreased urethral sphincter damage (6). Laser prostatectomy by Holmium laser enucleation of the prostate (HoLEP) has been introduced and promoted by Fraundorfer and Gilling over the last decade (7). High-powered holmium lasers have been used for the ablation and resection of the prostate due to their excellent incisional, ablative and hemostatic properties. The development of the transurethral tissue morcellator has allowed the rapid removal of prostatic tissue without size limitations on the prostatic gland. However, the HoLEP technique is difficult to master as it has a steep learning curve and longer surgery duration.

The 2-*μ*m (thulium) laser resection of the prostate-tangerine technique (TmLRP-TT) is a transurethral procedure that uses a thulium laser fiber to dissect whole prostatic lobes off the surgical capsule, similar to when dissecting a tangerine. Since 2006, >600 surgical cases have been carried out in the PLA General Hospital (Beijing, China). When compared with TURP, TmLRP-TT has a number of advantages, including improved spatial beam quality, more precise tissue incisions and the ability to operate in optional continuous or pulse-wave modes (8).

The objective of the present study was to evaluate the results for the clinical application of the 2-*μ*m continuous wave (cw) laser in transurethral vaporesection of the prostate.

## Patients and methods

### 

#### Patients

Between May 2007 and May 2008, a total of 115 symptomatic BPH patients from the PLA General Hospital were enrolled in the present study. This clinical trial was registered in PLA General Hospital, the Ethics committee approved this study and all patients signed a written informed consent form. All works were undertaken following the provisions of the Declaration of Helsinki. Their symptoms were evaluated according to the International Prostate Symptom Score (IPSS) and the quality of life (QoL) score. All patients underwent prostate-specific antigen (PSA), hemoglobin, urine and rectal examinations. The peak volume of the urinary flow rate (Qmax) and the post-void residual urine volume (PVR) were recorded and the patients with a PSA level of >4 *μ*g/l were subjected to a pre-operative prostate biopsy.

The patients were divided into 2 groups according to their prostate volume. The groups consisted of those with prostate volumes of ≤80 ml (n=85) and those with prostate volumes of >80 ml (n=75). The assessed outcomes of the present study were the test parameters of the patients and the surgical parameters of the procedures. These parameters were compared prior to and post-surgery within and between the two groups. The baseline characteristics of the patient data are presented in Table I.

The test parameter indices, including PVR, Qmax, IPSS and QoL, were also recorded prior to the surgery and at 6 and 12 months post-surgery. The blood sodium concentration (BSC) and the hemoglobin concentration (HGC) were also measured to assess each patient’s physical condition.

#### Equipment

A 2-*μ*m (thulium) laser with a rated output power of 70 W (LISA Laser Products OHG, Katlenburg-Lindau, Germany) was used in the cw mode throughout the procedure. The energy was delivered through 550-*μ*m RigiFib fibers. The laser fibers were introduced through a continuous flushing excision mirror. Saline irrigation was used in all the cases, with the surgery being observed via a television monitor.

### Surgical steps

**Figure 1:**
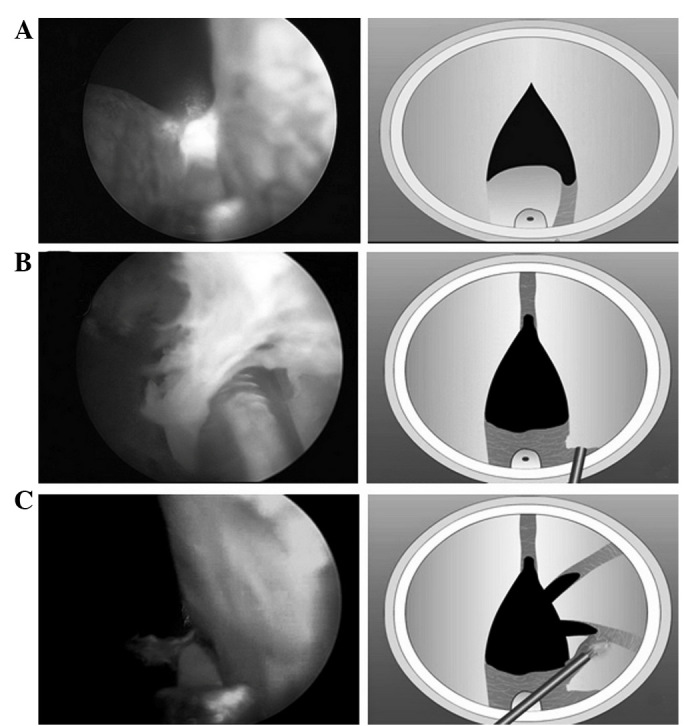
Surgical process of vaporization resection: left, operation video image; right, corresponding animation diagram. (A) Initial incision at 5 o’clock; (B) the left tip blunt dissection process; (C) resecting and segmenting left lobe.

*Step 1*.The surgical technique was similar to other resection procedures. Following initial incisions at 5 o’clock, 7 o’clock and 12 o’clock at the bladder neck, vaporesection of the median lobe was initiated (Fig. 1A).

*Step 2.* The fibre was moved semi-circumferentially from the verumontanum in the direction of the bladder neck, thereby undermining the tissue and cutting chips.

It was important to cut small sections that did not exceed the size of the inner sheath diameter so that the tissue sections were washed out without morcellation at the end of the procedure.

*Step 3.* Following the resection of the middle lobe of the prostate, the lateral lobes were removed in a retrograde fashion beginning at the verumontanum so that the external sphincter was preserved in the best way (Fig. 1B).

*Step 4.* Vaporesection of the lateral lobes was continued until in close proximity to the surgical prostatic capsule (Fig. 1C).

At the end of the procedure, the resected tissue chips were removed and a urinary catheter was inserted into the bladder. The suprapubic bladder puncture fistulation tubes were then removed.

#### Post-operative management

Peripheral blood samples were obtained from the patients to determine the BSC and HGC. The resected tissues were then weighed and urination and other data were recorded.

#### Statistical analysis

Data are presented as mean ± SD to compare the continuous variables of Qmax, IPSS, QoL and PVR prior to and post-surgery. P<0.05 was considered to indicate a statistically significant difference. The SPSS software package was used to perform the statistical tests. An analysis of correlation and regression equation was used.

## Results

**Figure 2:**
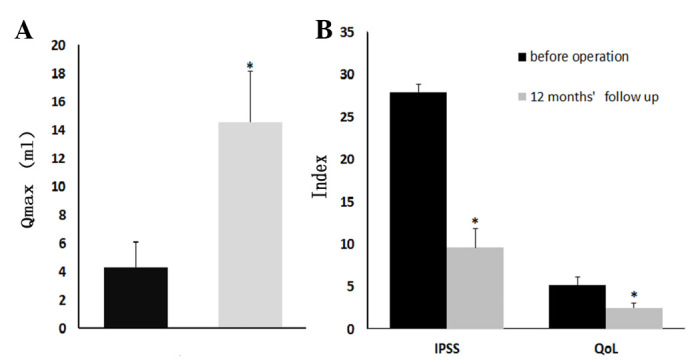
Statistical analysis result of patients with prostate volume over 80 ml. (A) comparison of maximum urinary flow rate before operation and 12-month follow-up. (B) comparison of IPSS and Qol (quality of life) index before operation and 12-month follow-up. ^*^P<0.05 compared with value before operation.

No significant bleeding or changes in the hematocrit were observed at any stage of the procedure, suggesting that the TmLRP-TT technique was an almost bloodless procedure. No intraoperative complications occurred. Compared with the baseline (pre-surgery) data, there were no significant improvements in the blood sodium and hemoglobin concentrations. In the group with a prostate volume of ≤80 ml, 23 patients required bladder flushing which ceased 24 h post-surgery. The median catheter indwelling time post-surgery was 3 days (range, 1–5 days). Acute urinary retention occurred in 5 patients 24 h subsequent to the removal of the catheter and emergency microscopic examinations revealed that this was caused by prostate tissue fragment obstructions. The median duration of hospitalization in these patients was 5 days (range, 1–7 days). A secondary hemorrhage occurred in 2 patients on the 25th and 28th day post-surgery. They each received a blood transfusion of 400 ml and following catheter indwelling and bladder flushing, the hemorrhage stopped. Stress urinary incontinence occurred post-surgery in 4 patients; they all recovered subsequent to receiving acupuncture and functional exercise treatments for 3 months. Anterior urethral strictures occurred in 2 patients at 3 months post-surgery; their emiction function recovered subsequent to receiving urethral dilation and urethroplasty separately. A post-operative histopathological examination identified that one of these patients had prostate cancer (Fig. 2).

In the patients with a prostate volume of >80 ml, there were significant improvements in the test parameters, including the IPSS, QoL scores, Qmax and PVR urine volume. Compared with the mean vaporization resection time of the ≤80 ml group (76.0±26.8 min), the resection time was longer at 95.0±13.2 min and the mean surgical clearance volume was 75.4±16.4 g (compared with 18.19±7.95). There was no sigificant difference in the post-operative urinary catheter indwelling time or the length of hospital stay in the two groups (shown in Table II). In total, 4 patients developed urge incontinence and 5 patients developed secondary anterior urethral strictures for which they all received the appropriate treatment. The test parameters of the two patient groups and the results of the 6-month and 1-year follow-ups are shown in Table II.

The statistical analysis results revealed that when compared with the data recorded pre-surgery, the IPSS, QoL score, Qmax and PVR values were significantly improved within each group (P<0.05), but that there was no statistically significant difference between the groups.

## Discussion

Advances in laser technology have led to the development of new laser treatments. HoLEP or green light laser photo-selective vaporization of the prostate (PVP) are new laser prostatectomies that require a shorter inpatient hospitalization period, cause fewer short-term adverse events and result in less bleeding than TURP (9). However, until the effectiveness of the holmium laser and the handling and safety properties of the green-light laser are combined, an ideal laser cannot be identified. The 2-*μ*m cw laser vaporesection of the prostate has been established as a new technology for the treatment of BPH (10–12). The RevoLix cw laser is a new surgical laser at a wavelength of 2013 nm or ∼2 *μ*m. The 2-*μ*m cw laser may have several advantages over other laser procedures as it has an improved spatial beam quality, operates in cw pulse modes, is capable of more rapid vaporization, makes precise incisions in the prostate tissue and uses normal saline as the irrigation fluid. The most significant difference is that its energy is delivered as a cw rather than a pulsed wave (13).

The surgeon uses the output end of the fiber in a reciprocating motion that follows a ‘U’ shape when operating the conventional 2-*μ*m laser; this technique is difficult with a steep learning curve, longer surgical time and a relatively low removal efficiency. This has been improved by split-vaporization resection of the prostate; this has the technical characteristics of a ‘W’-shaped superficial vaporization mark trench, multiple segmentation, a scanning type vaporization, a thin layer resection plus stripping and vaporization dressing of the wounds, resulting in an efficient and safe surgical mode.

Resection with the 2-*μ*m cw laser allows TURP-like chips of tissue to be cut and the resection time to be shortened as a result of using the simultaneous vaporization component of the laser. The efficient hemostasis and improved vision from the 2-*μ*m cw laser treatment of BPH may reduce bleeding and surgical risk. Therefore, the advantages of the 2-*μ*m cw laser vaporesection procedure are that it is a safe, efficient, bloodless and promising surgical procedure for the treatment of BPH (14). The experience and results gained by the present study from the 6- and 12-month follow-up of 155 patients showed that the vaporization resection method for treating BPH patients is reliably safe and that the post-operative indices, including the IPSS, QoL and Qmax, were significantly improved compared with those prior to surgery. These results are similar to other previous studies (15–17). Transurethral evaporation and visual laser ablation of the prostate were effective in relieving the symptoms of benign prostatic hyperplasia (18).

Strong biological tissue vaporization is the prominent characteristic of the 2-*μ*m laser (19). In this study, we acquired positive correlation between the collected prostatic tissue quality and the prostate gland specimen quality (data not shown), thus proved using 2-*μ*m laser enable manipulation and destruction of biological tissue of BPH with unprecedented precision and selectivity.

The most noteworthy findings in the present study were the observations that patients undergoing transurethral vaporesection of the prostate using the 2-*μ*m laser had a decreased rate of repeat surgeries and were 3 times more likely to have a decreased peak volume of urinary flow rate (7.2±2.7 versus 20.1±4.4, respectively, P<0.01), this shows the superiority of 2-*μ*m laser vaporesection in the treatment of BPH.

## Figures and Tables

**Table I:** Baseline characteristics data in patients with BPH.

	Prostate volume	
Characteristics	≤80 ml	>80 ml
Patients (n)	80	75
Patients with acute urinary retention (n)	12	9
Patients with bladder stones (n)	8	4
Mean prostate volume (ml)	63.4±13.6	123.7±26.7
Median age (years)	72.0±7.2	72.5±9.1
Median duration of urinary tract obstruction (years)	6.3±1.5	6.4±1.8

BPH, benign prostatic hyperplasia.

**Table II:** Statistical analysis results of TmLRP-TT in patients with benign prostatic hyperplasia (BPH).

	Prostate volume ≤80 ml			Prostate volume >80 ml		
	
Parameter	Pre-surgery	6 months	12 months	Pre-surgery	6 months	12 months
Test parameter						
PSA (ng/ml)	3.8±0.9	3.0±1.0	2.6±1.3			
Qmax (ml/sec)	7.2±2.7	18.9±3.5	20.1±4.4^a^	4.3±1.8	9.5±2.6	14.54±3.6^a^
IPSS	25.3±4.5	6.9±3.6	4.4±2.6^a^	27.9±6.5	12.3±4.2	9.6±2.3^a^
QoL	4.2±1.0	1.8±1.3	1.3±0.9^a^	5.2±0.5	3.1±0.8	2.5±0.6^a^
PVR (ml)	147.4±60.1	43.5±20.0	41.6±12.7^a^	115.3±35.2	38.9±16.7	32.0±15.1^a^
Surgical parameter						
Average time for vaporization resection (min)		76.0±26.8			95.0±13.2	
Average cutting prostate tissue volume (g)		18.19±7.95			75.4±16.4	
Post-operative urinary catheter indwelling time (days)		3.0±0.9			3.3±0.9	
Length of hospital stay (days)		3.9±1.6			4.8±1.8	

a Changed significantly compared with the results at hospitalization.

PSA, prostate-specific antigen; Qmax, maximum urinary flow rate; IPSS, international prostatic symptom scores; QoL, quality of life; PVR, post-voiding residual volume; BSC, blood sodium concentration; HGC, hemoglobin concentration; TmLRP-TT, 2-*μ*m (thulium) laser resection of the prostate-tangerine technique.
